# Summary Graphs Covary with Reading and Language Comprehension in School-age Children in the Spanish Language

**DOI:** 10.11621/pir.2024.0408

**Published:** 2024-12-15

**Authors:** Anabel Fernández-Blanco, Nancy Estévez-Pérez, Klency González Hernández

**Affiliations:** a University of Talca, Chile; b Cuban Neurosciences Center, Cuba; c University of Havana, Cuba

**Keywords:** reading comprehension, language, Speech Graph, text summaries

## Abstract

**Background:**

The standardized identification, psychoeducational assessment, and diagnosis of children at risk of reading comprehension (RC) difficulties is a highly specialized, time-consuming, and cumbersome process for teachers, psychologists, and researchers. Following the graph theory framework, text summaries, a ubiquitous RC measure used in schools, can be represented as networks of words (nodes) connected by arcs (TSGraphs). Do their resulting topological properties highlight individual variability in traditional reading/language comprehension measures?

**Objective:**

The objective of this study was to determine whether there is a significant association between individual variability in the connectivity measures of the TSGraphs of selected texts produced using graph theory and individual variability in traditional standardized measures of reading and language comprehension in Cuban school-age children.

**Design:**

Two correlational studies were conducted. Study 1 evaluated the association between the TSGraph properties and the reading comprehension of good and poor fifth-grade readers (N = 21). Study 2 evaluated the association between the TSGraph properties and language comprehension in sixth-grade children at risk of intellectual disability (IDr) and typically developing (TD) controls matched in age and gender (N = 42). Reading fluency, intellectual capacity, and vocabulary were controlled for in both studies.

**Results:**

Study 1 showed a significant association between TSGraph density and reading comprehension in fifth graders after controlling for reading fluency. Study 2 found that density significantly covaried with language comprehension in sixth graders after controlling for intellectual capacity.

**Conclusion:**

Topological measures of text summaries show promise for the assessment and characterization of reading and language comprehension. This is the first such study conducted on native Spanish speakers. Additional experimental studies in larger samples are required.

## Introduction

Reading comprehension (RC) is the ability to understand, use, and analyze texts to fulfill the reader’s purposes, develop knowledge, and participate in society (OECD, 2019; 2023). RC impacts varied academic areas (Oakhill, 2020; Smith et al., 2021; Vaughn et al.; 2015) and is considered a strong predictor of future academic success (Oakhill, Cain & Elbro, 2019; Silva & Cain, 2014). It influences cognitive processes such as problem-solving (McCarthy & McNamara, 2021), memory formation (Davidson et al., 2018; Mcnamara & Scott, 2001; Peng, 2018), and decision-making (Cartwright, 2015; Sun et al., 2018) and leads to devising effective and creative solutions to problems (Gick & Holyoak, 1983; Griffith & Lacina, 2017).

Difficulties in RC may slow the development of social and emotional skills and affect the individual’s self-esteem and self-concept (Cain, 2022; McCarthy & McNamara 2021). In Cuba, González and collaborators (2016) reported RC difficulties in more than 20% of the fifth- to sixth-grade children assessed in their study. Hence, identifying children at risk or presented with RC difficulties is key to providing the available remediation/intervention resources and orientation to the children, teachers, and families.

Several theoretical models of RC have been proposed to elucidate this complex process (see Sanır & Özmen, 2022; for a review). The main components of most comprehension models are reading fluency, prior knowledge, vocabulary, reading comprehension strategies, inference, and motivation (Sanır & Özmen, 2022). Additionally, other complex cognitive processes including intellectual capacity and working memory influence RC (Smith et al., 2021). Hence, the standardized identification, psychoeducational assessment, and diagnosis of children at risk of RC difficulties is a challenging and time-consuming process that requires specialized personnel.

Different types of RC assessments have been used in pedagogical and experimental scenarios, including free recall, cued recall, cloze/multiple choice/true-or-false questions, sentence recognition, reenactment, and summaries (Smith et al., 2021; Duke et al., 2021; Bogaerds-Hazenberg et al., 2020). This methodological variability reflects the lack of consensus regarding the comprehension outcomes that could/ should guide teachers in the classroom and, hence, the best methods for measuring these outcomes (Smith et al., 2021).

RC assessment using text summaries affords an ecological alternative for teachers/researchers in the classroom or parents at home. Learning to summarize texts is a skill acquired through schooling as it is useful in any academic domain. Summaries are considered a surface level representation of the reader’s recollection of the literal aspects of texts (the literal, propositional representation of a text held in the working memory or textbase, according to Kintsch & Van Dijk, 1978). The ability to summarize information is of great pedagogical interest. However, RC assessment using text summaries relies on the subjectivity/expertise of the evaluators despite the availability of rubrics, and it is a laborious undertaking owing to the difficulty of giving detailed feedback on every summary, especially in large classes.

New developments in data mining and complex systems analysis allow us to treat texts as measurable structures. Specifically, graph theory offers a systemic analysis of the structural characteristics of recall-based reports to quantify cognitive deficits (Bertola et al., 2014; Mota et al., 2012; 2014). In these studies, discourse is represented by a graph where the words are represented by nodes and the temporal relationships between consecutive words are represented as axes or arcs. It is possible to calculate the general attributes, or properties, of the resulting “speech graph” and interpret these as parameters that provide information on the processes underlying the production of discourse/texts.

The quantitative properties produced by speech graph analysis can be classified into general, recurrence, and global measures (Mota et al., 2012; Palaniyappan et al., 2018). General properties are represented by the number of nodes and arcs (the number of different words and the connections between words in a text, respectively) and reveal the lexical diversity and language cohesion/articulation of the text or discourse. Recurrence measures are sequences of one to three different words indicating sequences that are revisited. Global measures such as density (number of directly connected words divided by all possible connections between words) and the largest connected component (LCC) (the number of nodes in the maximal component in which all pairs of nodes are reachable from one another, indicating how well connected the words of the text are) are associated with the connectivity of the network and therefore with discourse cohesion.

This approach is particularly valuable in clinical or psychoeducational settings, where traditional assessments may fail to capture the nuances of disorganized speech/text production patterns. Speech graphs have been used to reveal cognitive deficits in pathological populations such as patients with obsessive–compulsive disorder (OCD) (Gomes et al., 2023), psychosis (Mota et al., 2012; Palaniyappan et al., 2018, Spencer et al., 2020), or dementia (Bertola et al., 2014). The studies found that the dream-based reports of psychotic patients are less interconnected than those of the control subjects, similar to OCD patients who exhibit significantly lower lexical diversity, lower speech connectedness, and a higher recurrence of words (Gomes et al., 2023). Additionally, a negative correlation was found between connectivity measures (global measures associated with network connectivity and therefore discourse cohesion) and the symptoms, indicating that the less coherent the speech production, the more cognitively damaged the subject (Mota et al., 2014).

In the case of dementia, graph theory applied to tests of verbal fluency allowed a correct classification of subjects with Alzheimer’s Disease and cognitive impairment (Bertola et al., 2014). The cognitive damage was proportional to the increase in graph density, the reduction in diameter, and the average decrease in the shortest path length.

Additionally, graph theory was used to explore the relationship between episodic memory reports and academic achievement, also including cognitive measures such as IQ and theory of mind (Mota et al., 2016). Speech parameters covaried with cognitive measures and with reading performance, namely, the number of words (nodes), the connections between words (arcs), and the minimum count of word–word association repetitions (repeated arcs). Children with a higher performance in intellectual capacity tasks, theory of mind, and reading reported episodic memory events with richer vocabulary and higher speech articulation.

More recently, two studies investigated the relationship between speech graph properties and relevant variables in language production. The first study (Lemke et al., 2021) explored bilingual Portuguese–English-speaking children, revealing a correlation between graph attributes (i.e., connectedness measured by the number of nodes and edges, LCC, and text density) and the levels of syntactic complexity in both languages, demonstrating that, as children develop more complex writing strategies in Portuguese, they progress in written English to the same extent. The second study (Botezatu et al., 2022) focused on second language learners of Spanish and Chinese, examining the impact of lexico-semantic processes on the connectedness (measured by the number of nodes inside the LCC) of narratives. The results indicated a significant positive correlation between connectedness and speech production measures in second language (L2)-Spanish and L2-Chinese learners.

These studies suggest that speech graph properties are useful when investigating the typical development of complex cognitive processes such as memory and language and, potentially, reading comprehension. Speech graph analysis seems a suitable and robust framework for understanding the organization and coherence of spoken or written language by focusing on how speech/texts are structured rather than on their content (without needing to interpret the meaning behind the words produced). Hence, the following question arises: Is it possible to quantitatively measure the properties of text summaries using graphs (TSGraphs) as a proxy for individual variability in the reading and language comprehension of school-age children?

The present investigation aims to determine whether there is a significant association between individual variability in the connectivity measures of the TSGraphs of selected texts produced using graph theory and individual variability in traditional and standardized measures of reading and language comprehension in Cuban school-age children. We hypothesize that there exists a statistically significant association between the variability in general and global measures of connectedness in the TSGraphs and the individual variability in standardized measures of reading and language comprehension.

To test this hypothesis, two studies were conducted to more comprehensively evaluate the comprehension processes implicated in both reading and language tasks. The first study examined the association between a standardized measure of reading comprehension and the TSGraph topological properties calculated from summaries produced by fifth-grade poor and good readers. Since children at risk of intellectual disability are more likely to exhibit lower language performance (Adlof et al., 2017; Di Blasi et al., 2019), the second study examined the association between a standardized measure of language comprehension and the properties of the TSGraphs of summaries produced by sixth-grade children exhibiting the risk of intellectual disability and typically developing pairs matched in grade and gender.

To the best of our knowledge, no speech graph studies have been conducted on native Spanish speakers to explore the feasibility of using the topological properties of summaries, understood as speech graphs, to determine individual differences in reading and language comprehension in school-age children. Hence, this is an opportunity to contribute data to this yet unexplored field and contrast it with previous reports on English, Portuguese, L2-Spanish, and L2-Chinese (Bertola et al., 2014; Botezatu et al., 2022; Lemke et al., 2021; Mota et al., 2012, 2014, 2016; Coelho et al., 2018). The results will enhance automated psychoeducational assessment and psychiatric screening/diagnostic accuracy by establishing a reference for developing a cost-effective, scientifically driven alternative to assess summaries, relevant to the population at risk of learning disabilities.

## Methods

### Participants

The sample was recruited as part of the KHE PhD project. It comprised 63 school-age children: 21 (11 girls) fifth graders and 42 (21 girls) sixth graders (see sample details in Table 1). The children were assessed by trained psychology students in a well-lit and quiet room in their schools.

**Table 1:** Sample description

5^td^ Grade (N=21, F=11)				
WM Index	Vocabulary Index		Fluency Index	
M (SD)	M (SD)		M (SD)	
.60 (.10)	37.10 (12.10)		103.40 (30.60)	
6^th^ Grade (N=42, F=21)				
	Attention (d2)		Vocabulary Peabody Raw Score	Intellectual Capacity RIST Index
TP M (SD)	FR M (SD)	CP M (SD)	M (SD)	M (SD)
33.05 (78.06)	112.48 (25.01)	17.02 (6.43)	102.07 (0.27)	72.38 (17.31)

*Note: M: Mean, SD: Standard Deviation, WM: Working Memory, TP: Total Performance, CP: Concentration Performance, FR: Fluctuation Rate*

### Procedure

Two studies were conducted. In each study, groups of children with difficulties in reading comprehension and intellectual capacity/language comprehension were evaluated. Concurrent assessments using standardized comprehension tests and summaries of age-appropriate texts were conducted. The selected texts had a difficulty level appropriate for fifth and sixth graders, according to Inflesz 1.0 software (González & Estévez, 2019). This program evaluates the readability of texts written in Spanish by considering nine parameters (number of words, syllables, phrases, relationships between them, etc.) and classifies the texts into five difficulty levels (very difficult, quite difficult, normal, quite easy, and very easy). Cognitive assessments were performed to control for variables that covary with reading comprehension and academic performance, such as executive functions (Cortés Pascual et al., 2019), vocabulary (Schmitt & Schmitt, 2020), intellectual capacity (Blakemore & Bunge, 2012), reading fluency (Stanley, Petscher & Catts, 2018), and attention (García-Madruga et al., 2012).

### Study 1: TSGraph properties and reading comprehension

Fifth-grade children’s reading comprehension was evaluated using the raw score obtained in the Reading Comprehension Assessment Test (PECL, from its acronym in Spanish: “Prueba para Evaluar la Comprensión Lectora”) (Ferreres et al., 2009) text “El Rebelde”. Using this test as a golden rule, children were classified into good and poor readers. The fifth-grade children produced a summary of the text “El relicario”. The text was given to the children to read independently and without time restriction. Then, they were asked to summarize the text without time restriction. Intellectual capacity was assessed using the percentile on the Raven’s Coloured Progressive Matrix Test (Raven, Court & Raven, 1993). The included children ranged between the 25th and 95th percentiles and met the criteria of not having repeated any grade. In this group, a reading fluency index was calculated using a reading fluency test (Mosquera, 2011), multiplying the total number of words correctly read in the text by 60 and dividing the resulting value by the total reading time in seconds. A working memory index, assessed using a working memory task, was calculated as the ratio between the individual score/maximum possible score (McInerney, Hramok & Kerns, 2005), and an individual vocabulary measure, the individual raw score in the Vocabulary Subtest of the WISC-R (Wechsler, 1974), was included.

### Study 2: TSGraph properties and language comprehension

Sixth-grade children at risk of intellectual disability (IDr) and a group of typically developing (TD) controls were compared. Language comprehension was evaluated using the free recall, recognition, and total raw scores obtained in the Narrative Memory subtest from the NEPSY II Battery (Korkman, Kirk & Kemp, 2014). The children produced a summary of the text “Japón, primero en dibujos animados”. The text was given to the children to read independently and without time restriction. Then, they were asked to summarize the texts without time restriction. Intellectual capacity was assessed using the RIST Index of the Reynolds Intellectual Screening Test (RIST; Reynolds & Kamphaus, 2013). Vocabulary was assessed using the raw score in the Peabody Vocabulary Test (Manzano et al., 2003). Finally, attention was assessed using the Concentration Endurance Test (d2) (Wassenberg et al., 2008) with its three related indicators: Total Performance (TP), Concentration Performance (CP), and Fluctuation Rate (FR).

### TSGraphs

SpeechGraph software (https://www.neuro.ufrn.br/softwares/speechgraphs) was developed by the Federal University of Rio Grande do Norte (Brazil) in 2012. This tool represents a text as a graph (G): the words (w) of the text are represented as nodes (N) and the connections between the words are represented as edges (E). G = (N, E), with N = {w1, w2, w3,…} as the set of nodes and E = {(wi, wj)} as the set of edges between words wi and wj in N (see the definition of the output parameters in *Appendix*). In both studies conducted, graph attributes were calculated using the whole text.

### Statistical Analysis

Descriptive statistics of the data were produced using the STATISTICA program (StatSoft, Inc., 2007; version 8.0., www.statsoft.com). The univariate normality of data was evaluated by examining skewness and kurtosis, with absolute values of skewness lower than 2 and kurtosis lower than 7 considered low departures from normality (Bryne, 2010). Most variables met these criteria except for density (skewness = 2.47 and kurtosis = 6.54) in Grade 6, and RE (skewness = 2.57 and kurtosis = 7.99) and PE (skewness = 2.27 and kurtosis = 6.66) in Grade 5. For these variables, a logarithmic transformation (log10) was applied to achieve a normal distribution. However, the skewness and kurtosis did not improve; therefore, nonparametric alternatives were used in the corresponding analyses.

To control for the possible contribution of domain-general cognitive variables to variability in reading/language comprehension and academic performance, correlations were performed between the corresponding variables. Taking into account the small sample size in the fifth-grade group, partial correlations were conducted between the summaries’ properties and reading comprehension, controlling for domain-general cognitive variables exhibiting significant correlations. In the sixth-grade sample, following the correlations analysis, a multivariate linear regression analysis was conducted to identify the general cognitive processes explaining a significant proportion of variance in language comprehension. Additional simple regression analyses were conducted to calculate the residuals of the dependent variable and include them in the correlations with the syntactic properties of summaries.

Finally, the independent-samples Student’s t-test and Mann–Whitney U test were conducted to compare the syntactic properties between the children classified as good/poor readers and IDr/controls (significance level of 0.05).

To address multiple comparisons the false discovery rate (FDR) correction method was applied. This approach is particularly suited for studies with many comparisons involving multiple psychological variables, such as the present studies. The FDR provides a balance between controlling for false positives and maintaining statistical power. The adjustments were performed using the Multiple Test Correction Tool (https://multipletesting.com/analysis), as implemented by Menyhart, Weltz, and Győrffy (2021).

## Results

### Descriptive statistics for both studies

The descriptive statistics regarding domain-general cognitive processes and the academic performance of fifth graders are in *Table 2*. Results of the domain-general cognitive processes and language assessment of sixth graders are presented in *Table 3*.

**Table 2:** Descriptive statistics of the domain-general cognitive processes and academic achievement of 5th-grade children

Measures	All Sample (N=21) Mean (SD)	Minimum Value	Maximum Value
**Academic Achievement**			
Spanish Language Achievement RS	45.69 (4.41)	32.30	50.00
Reading Achievement RS	17.42 (1.75)	14.00	20.00
Writing Achievement	18.05 (1.56)	15.00	20.00
**Cognitive Processes**			
Working Memory Index	.56 (.08)	.44	.69
Reading Fluency Index	103.44 (30.63)	61.27	175.81
WISC Vocabulary RS	37.14 (12.07)	16.00	62.00

*Note: M: Mean, SD: Standard Deviation, RS: raw score*

**Table 3:** Descriptive statistics of the domain-general cognitive processes of 6th-grade children.

Variables	All Sample (N=42) Mean (SD)	Minimum Value	Maximum Value
**Cognitive Processes**			
RIST Index	72.38 (17.31)	48.00	115.00
Attention (d2) – TP	233.05 (78.06)	116.00	475.00
Attention (d2) – CP	112.48 (25.01)	76.00	217.00
Attention (d2) – FR	17.02 (6.43)	6.00	35.00
Vocabulary (Peabody)	102.07 (10.27)	88.00	126.00

*Note: M: Mean, SD: Standard Deviation, TP: Total Performance, CP: Concentration Performance, FR: Fluctuation Rate*

In the fifth-grade group, 9 out of 21 children (42.86%) were identified as being at risk of intellectual disability (below the 50th percentile). However, the independent-samples Student’s t-tests indicated that children at risk exhibited similar performance compared to their typically developing peers in all cognitive tasks, including the verbal reasoning WISC subtest and the text summary quantitative measures calculated using the SpeechGraph (see details in *Table 4*).

**Table 4:** Independent-samples t-tests comparing 5th-grade children at risk of intellectual disability (IDr) and typically developing (TD) controls.

Cognitive measures	IDr Children (N=9) M (SD)	TD Children (N=12) M (SD)	t(p)
Working Memory Index	.59 (.06)	.55 (.09)	1.18 (.25)
Reading Fluency Index	92.85 (31.67)	111.39 (28.56)	–1.41 (.18)
WISC Vocabulary RS	34.33 (13.74)	39.25 (10.78)	–.92 (.37)
TextoRebelde_ PECL	6.44 (2.30)	6.25 (2.05)	.20 (.84)
**Graph metrics**			
WC	75.00 (32.16)	97.75 (48.28)	–1.22 (0.24)
Nodes	52.67 (18.41)	65.42()25.85	–1.26 (0.22)
Edges	73.67 (32.05)	96.17 (48.07)	–1.21 (0.24)
RE	2.22 (2.82)	4.25 (5.19)	–1.06 (0.30)
PE	2.56 (2.88)	4.75 (5.17)	–1.14 (0.27)
L2	.33 (.50)	.00 (.00)	–.55 (.59)
L3	1.78(1.56)	.50 (.80)	–.96 (.35)
LCC	52.67 (18.41)	2.58 (2.11)	–1.26 (.22)
LSC	48.78 (18.91)	65.42 (25.85)	–1.11 (0.28)
ATD	2.72 (0.27)	60.42 (26.62)	–1.18 (0.25)
Density	.06 (0.02)	2.88 (.34)	1.07 (.30)
Diameter	9.00 (1.66)	.05 (.02)	–.94 (.36)
ASP	4.03 (.39)	10.08 (3.15I	–.22 (.83)
CC	.05 (.05)	4.09 (.71)	.16 (.88)

*Note: SD: Standard Deviation, ID: Intellectual Disability*

### Study 1: TSGraph Properties and Reading Comprehension

#### Correlations between Domain-General Cognitive Processes and Reading Comprehension

The fifth-grade sample showed a statistically significant covariation between the reading fluency index and reading comprehension (r = .66, r^2^ = .44, t(21) = 3.88, p = .001). After applying the FDR correction for multiple comparisons (the critical p-value was adjusted to .002), this result remained significant. No statistically significant correlations were found between working memory, vocabulary, and reading comprehension in this sample.

#### Correlations between Graph Properties and Reading Comprehension

The correlations between all graph attributes and reading comprehension were analyzed. Only density showed a significant covariation (r = –.64, r^2^ = .41, t(21) = –3.66, p=.002). This result remained significant after the FDR correction for multiple comparisons.

**Figure 1.**
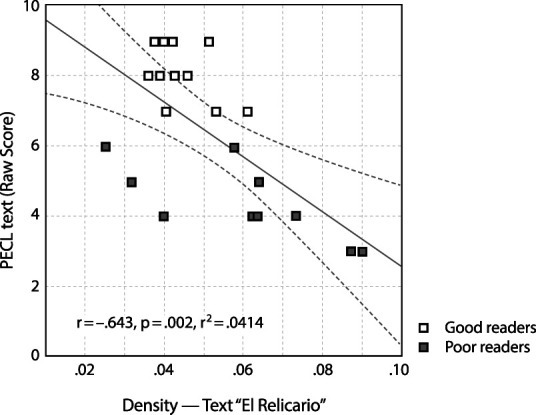
Linear correlation between density and reading comprehension (PECL_Raw Score).

Considering the statistically significant association observed between reading fluency and reading comprehension, as well as between the graph-derived density attribute and reading comprehension, a partial correlation was conducted between the TSGraph density and reading comprehension, controlling for the effect of reading fluency. The result revealed a negative, statistically significant correlation between the raw scores for reading comprehension and density (r = –.45, r^2^= .21, t(21) = –2.16, p = .045) (see *Figure 1*; note that the linear correlation shown is not corrected for the effect of reading fluency).

A closer examination of individual summaries confirmed that poor readers’ summaries included details and information not directly related to the central idea of the text (see *Appendix* for a translation of the texts to English).

#### Comparison of Academic Achievement and Domain-General Cognitive Processes in good vs. poor readers

After applying the FDR correction for multiple comparisons (compared variables: academic achievement, domain-general cognitive processes, and TSGraph properties), the new critical p-value was set to p < .014. Comparisons with p-values lower than this threshold are subsequently considered statistically significant.

The independent-samples t-test showed that good readers exhibited significantly higher results in all academic achievement variables compared to poor readers. After the FDR correction for multiple comparisons, the differences in reading and writing achievement were maintained. In the case of cognitive processes, a statistically significant difference was observed only in the reading fluency index, even after the FDR correction for multiple comparisons (see *Table 5*).

**Table 5:** Comparison of academic achievement, domain-general cognitive processes, and syntactic properties between good and poor readers

Measures	Good readers (N=11) Mean (SD)	Poor readers (N=10) Mean (SD)	t(p)
**Academic Achievement**			
Spanish Language Achievement RS	48.32 (1.97)	42.80 (4.60)	3.64 (.002)
Reading Achievement RS	18.55 (1.04)	16.20 (1.55)	4.12 (.001)
Writing Achievement	18.82 (1.47)	17.20 (1.23)	2.72 (.014)
**Cognitive Processes**			
Working Memory Index	.60 (.08)	.53 (.06)	2.12 (.047)
Reading Fluency Index	120.74 (29.90)	84.42 (18.01)	3.33 (.004)
WISC Vocabulary RS	38.82 (13.70)	35.30 (10.38)	.66 (.519)

*Note: RS: Raw Scores*

#### Comparison of TSGraph properties in good vs. poor readers

**Figure 2.**
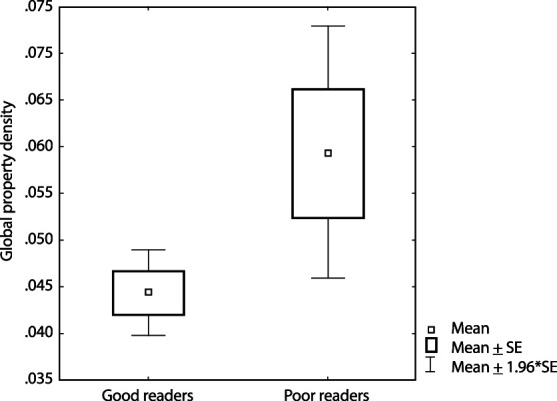
Box plot representation of the TSGraph attribute density in good and poor readers.

The independent-samples Student’s t-test indicated that good readers exhibited a significantly lower graph density (t (19) = 2.14, p = .046; M = .045, SD = .007) compared to poor readers (M = .059, SD = .021, see *Figure 2*). While this result was statistically significant before applying the FDR, with a critical p-value of.014, it no longer reached statistical significance. No significant differences were found between the subgroups in any of the remaining syntactic properties of the summaries.

### Study 2: TSGraph properties and language comprehension

#### Correlations between Domain-General Cognitive Processes and Language Comprehension

Significant Pearson correlations were found between language comprehension and the RIST Index (r = .46, r2 = .21, t(42) = 3.34, p = .002) and vocabulary (r = .36, r2 = .13, t (42) = 2.48, p = .017). After applying the FDR correction (critical p- value = .017), these results remained significant.

The multivariate regression analysis showed that only variability in intellectual capacity had a statistically significant effect on the dependent variable (RIST Index: SS = 224.71, df = 1, MS = 224.71, F = 4.32, p = .044, partial eta-squared = .0996, observed power = .526; Peabody RS: SS = 3.71, df = 1, MS = 3.71, F = .071, p = .79, partial eta-squared = .0018, observed power = .058).

#### Correlations between TSGraph properties and Language Comprehension

**Figure 3.**
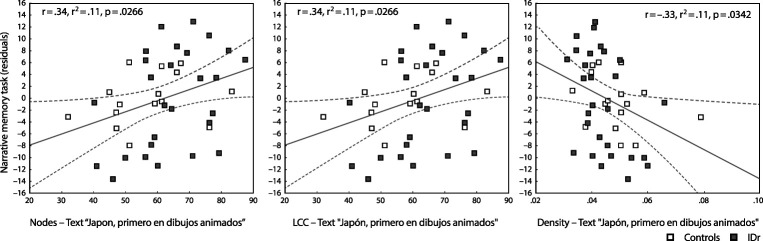
Linear correlation between TSGraph properties and language comprehension.

The correlations between the properties of the summary graphs of sixth-grade children derived from the text “Japón, primero en dibujos animados”, and the residuals of the regression between language comprehension and intellectual capacity yielded statistically significant covariations with the number of nodes (r = .34, r^2^ = .12, t(42) = 2.30, p = .026), the largest connected component (r = .34, r^2^ = .12, t(42) = 2.30, p = .026), and the density of the summary (r = –.35, t(42) = –2.36, p = .023). The correlation associated with density was calculated using Spearman’s rank correlation, as this variable did not meet the normality assumption. After the FDR correction for multiple comparisons was applied (the critical p-value was adjusted to .023), only the correlation with density remained significant. However, although the correlations with the number of nodes and the largest connected component showed a clear trend, these did not remain statistically significant (see *Figure 3*).

#### Domain-General Cognitive Processes and syntactic properties between IDr and TD groups

As expected, the 27 sixth graders (64.29%) at risk of ID (RIST index < 80) showed significantly lower results in most of the cognitive measures (see *Table 6*). Notably, the majority of these measures remained statistically significant after applying the FDR correction (critical p-value = .034).

**Table 6:** Independent-samples t-tests comparing 6th-grade children at risk of intellectual disability (IDr) and typically developing (TD) controls.

Cognitive measures	IDr Children (N=27) M (SD)	TH Children (N=15) M (SD)	t(p)
Narrative Free Recall Memory RS	20.59 (9.11)	28.93 (5.55)	–3.22 (.003)
Narrative Free Recall Memory SS	17.78 (2.01)	19.00 (.00)	–2.35 (.024)
Narrative Free+Guided Memory Recall RS	24.89 (8.40)	31.60 (4.87)	–2.83 (.007)
Narrative Free+Guided Memory Recall SS	14.96 (3.59)	17.53 (1.68)	–2.61 (.013)
Narrative Recognition Memory RS	13.44 (1.97)	14.60 (1.24)	–2.05 (.047)
Narrative Total RS Memory	38.33 (9.89)	46.20 (5.93)	–2.80 (.007)
RIST Index	61.30 (9.02)	92.33 (7.92)	–11.14 (.000)
TP	260.19 (79.50)	184.20 (46.13)	3.39 (.002)
CP	106.44 (26.38)	123.33 (18.52)	–2.19 (.034)
FR	17.74 (6.32)	15.73 (6.65)	0.97 (.339)
Peabody Verbal Test RS	97.96 (7.96)	109.47 (10.01)	–4.09 (.000)

*Note: RS: Raw Scores, SS: Standard Scores*

The independent-samples Student’s t-test comparing the subgroups’ TSGraph properties showed no statistically significant differences in any of them (Nodes: t(40) = -1.83, p = .075) IDr Children: M = 64.29 SD = 12.22, Controls: M = 56.93 SD = 13.05; LCC: t(40) = -1.83, p = .075 IDr Children: M = 64.29 SD = 12.22, Controls: M = 56.93 SD = 13.05). The non-parametric independent sample comparison conducted on density was the same (Rank Sum TD Children = 382.5, Rank Sum IDr Children = 520.50 U = 142.50, Z = -1.56, p = 0.12).

## Discussion

The results of these studies suggest that the summary density of grade-appropriate texts is informative regarding the individual variability in standardized measures of reading and language comprehension in both fifth- and sixth-graders’ samples. Note that these studies were conducted in different grades, using different measures of information processing and comprehension. Moreover, different statistical procedures controlling for the effect of domain-general cognitive factors were used, and yet consistent results were found, in line with previous reports.

In both samples, density was informative and showed a negative association with reading and language comprehension. Additionally, poor readers showed significantly higher density values. Therefore, the higher the density of the summary, the worse the cohesion and organization of ideas. As suggested by this negative correlation, poor readers’ summaries included details and information not directly related to the central idea of the text.

A previous study discriminating degrees of severity of cognitive impairment through verbal fluency, evaluated using the SpeechGraph properties (Bertola et al., 2014), found that patients with more severe symptoms showed higher density values for the derived networks, as well as lower values for the average shortest path (ASP). Both properties, together with the word count, nodes, edges, and diameter, discriminated with adequate sensitivity and specificity between the clinical sample and controls. Control subjects displayed less dense networks than patients with cognitive impairment.

Another study reported that density, understood as an index of linearity in discourse (Coelho, Mattos & Tannock, 2018) and used as a measure of narrative efficiency, showed the greatest difference between patients identified with attention-deficit/ hyperactivity disorder and controls. The latter showed significantly lower values of the speech graph’s density. The results presented here are in line with those of Bertola et al. (2014) and Coelho, Mattos & Tannock (2018).

Moreover, in relation to the construction–integration model (Kintsch & Van Dijk, 1978), our results suggest that an atypically structured textbase could be implicated in low reading and language comprehension. The construction–integration model posits that reading comprehension stems from the interactions between the literal, propositional representation of a text held in working memory (the textbase) and the reader’s preexisting schemata contained in long-term memory (Kintsch & Van Dijk, 1978), which forms a representation of the meaning of the text (the situation model).

For most readers, the textbase is automatically constructed and requires little conscious effort. In contrast, poor readers are believed to construct a less detailed situation model as compared to fluent readers, putatively due to a less coherent text-base and/or less developed schemata (Kintsch, 1998, Smith et al., 2021). The TS-Graphs can be interpreted as a suitable structural representation of the textbase and may advance our understanding of the cognitive underpinnings of comprehension processes in the context of both reading and communication.

Additional global attributes showed statistically significant covariations before the FDR multiple comparison correction and did not maintain them after the correction; nevertheless, given the low sample size and the stable results obtained in the same sample for the rest of the variables, we will briefly discuss the implications of the trends in the sixth-grade sample toward significant correlations in the case of nodes and largest connected component with reading comprehension. In these cases, the correlations between the previously mentioned attributes and language comprehension were positive and direct. These results are also in line with previous findings. Nodes, which indicate the lexical diversity of the summaries, were positively correlated with language comprehension even though the length of the studied summaries ranged between one and three paragraphs and were considered short texts (Brown, 2018). Nodes were also reported to correlate positively with IQ, which included language skills in Mota et al. (2016).

The LCC (and LSC) properties have also been reported to directly covary with language comprehension. In the study of the relationship between the structure of autobiographical memories and cognitive and reading performance (Mota et al., 2016), LCC and LSC were the properties more stably correlated with reading competence at different moments of the investigation. The authors found a similar direct association between these properties in all cases, despite several differences between the studies.

In Mota et al. (2016), the text input to SpeechGraph was the transcription of autobiographical memories, based on reports of long- and short-term declarative memory. Here, the task consisted of writing a summary of a narrative text, whose length could hinder the ability to evoke related details. It may be easier to establish connections between known events that are directly retrieved from memory than from the content of a narrative text that does not necessarily refer to the child’s experience. The chance of producing a longer, varied, and interconnected text favors oral discourse (McCarthy & McNamara, 2021; Roberts & Street, 2017). Additionally, from the executive perspective, organizing a written text requires greater attention and planning resources than oral discourse (Cain, 2022; Ellis, 2016; Smith et al., 2021). However, similar correlation sizes were found in both studies: cognitive performance (IQ and theory of mind (ToM) performance) and nodes (IQ: r =0.36, p =.0014; ToM: R =0.35, p =.0022) and LCC (IQ: r =0.40, p =.0005; ToM: r =0.34, p =.0023). These similar results suggest that children who employ a larger number of different words, make more connections among them, and have fewer repetitions of word–word associations (in either spoken or written discourse) performed better on IQ, ToM, and language comprehension tests.

Regarding school achievement, Mota et al. (2016) also found significant positive correlations between reading performance and LCC (r =0.33, p =.0041), consistent with the above-discussed result. LCC was also directly correlated with reading, even after controlling for the effect of IQ and ToM.

The study conducted by Lemke et al. (2021) of bilingual Portuguese–English-speaking children demonstrates how levels of syntactic complexity in writing are mirrored by increases in graph attributes such as connectedness and text density. Similarly, the present results reveal associations between topological measures such as graph density and reading comprehension in native Spanish-speaking fifth and sixth graders. This correspondence suggests the plausibility of generalizing the application of graph theory principles across diverse linguistic contexts and tasks, despite the difference in task demands—speech production in Lemke’s case and reading comprehension in our studies. Such parallelism reinforces the plausibility of graph theory as a framework for understanding language processing mechanisms, transcending specific linguistic modalities and tasks.

In general, the results reported here suggest that evaluations based on quantitative measures derived from graph theory provide a suitable framework for a more in-depth analysis of the impact of syntactic variables, especially topological attributes related to reading comprehension, and SpeechGraph attributes to differentiate between clinical samples. The inability to produce a text with distinctive properties such as cohesion (which can be quantified through density) or a specific connectivity pattern (captured by LCC) can be a predictor of low reading/language comprehension. Identifying this will contribute to improving the precision of evaluation systems and the effectiveness of intervention and follow-up strategies for subjects at risk of learning/language difficulties hampering reading and language comprehension.

## Conclusion

The quantitative properties/attributes of text summaries treated as graphs can indicate the individual variability in standardized language and reading comprehension measures in school-age children. They support generalizing the application of graph theory principles across diverse linguistic contexts and tasks. Further research should explore including these measures in automated evaluation systems that identify and stimulate reading/language comprehension processes and/or the impact of different intervention strategies. They can be applied to larger samples of typically developing children and children at risk of language/reading/learning disabilities or low academic achievement. To the best of our knowledge, this is the first study using the TSGraph properties to explore reading/language comprehension in the Spanish language.

## Limitations

The preliminary results presented here should be interpreted with caution, owing to the small sample size of the groups compared and the fact that, in Study 1, a high percentage of children at risk of low intellectual capacity were identified (approximately 42% were classified in the 25^th^ percentile in the Raven Test). These results were unexpected, considering that the sample was made up of children who had already passed at least four grades of the general education system. It should be taken into account that the children were classified using foreign norms (Chilean in the case of the Raven Test). This could explain the discrepancy between the children’s intellectual performance and academic achievement, particularly since they exhibited statistically similar results to the children in the ≥50th percentile in the rest of the cognitive and academic assessments conducted. Finally, please note that most of the research on SpeechGraphs has been conducted on oral, not written, narratives; hence, interpretation of these preliminary results should also consider this factor.

## Appendix

**Table:** Variables derived from the SpeechGraph (outputs)

Syntactic of the summary properties	Operational definition
**General Properties: quantification of the main elements that make up the network.**	
**Word Count: WC**	Number of words in the text.
**Number of nodes: N**	Number of different words in the text.
**Number of Edges: E**	Number of links between words in the text.
**Recurrence Properties: quantification of the repetition of the edges between words and patterns of repeated words.**	
**Repeated Edges: RE**	Sum of all edges linking the same pair of nodes.
**Parallel Edges: PE**	Sum of all parallel edges linking the same pair of nodes (connections between two words) in opposite directions.
**Loop of one node: L1**	Sum of all edges linking a node with itself.
**Loop of two nodes: L2**	Sum of all loops containing two nodes (sequences of two different words).
**Loop of three nodes: L3**	Sum of all loops containing three nodes (sequences of three different words).
**Connectivity Properties: quantification of the number of words connected through paths of edges, regardless of directionality.**	
**Largest Connected Component: LCC**	Number of nodes in the maximal component in which all pairs of nodes are reachable from one another in the underlying undirected subgraph (an indicator of how well connected the words of the text are).
**Largest Strongly Connected Component: LSC**	Number of nodes in the maximal component in which all pairs of nodes are reachable from one another in the directed subgraph (also, an indicator of how well connected the words of the text are).
**Global Properties: quantify the topological characteristics of complex graphs.**	
**Average Total Degree: ATD**	Given a node n, the Total Degree is the sum of “in and out” edges. The Average Total Degree is the sum of the Total Degree of all nodes divided by the number of nodes (how many links the word has with any other words).
**Density**	Number of edges divided by possible edges (D = 2*E/N*(N-1)), where E is the number of edges and N is the number of nodes (number of directly connected words divided by all possible connections between words).
**Diameter**	Distance, in number of nodes, from the connection between the most distant pair of connected nodes in the text.
**Average Shortest Path: ASP**	Average of the shortest paths between each pair of nodes in the text.
**Average Clustering Coefficient: CC**	Given the node *w1*, the clustering coefficient is the measure of how many nodes are directly connected to the node *w1*, being also directly connected to each other in a neighborhood; the average clustering coefficient is the sum of the clustering coefficients for each node divided by the number of elements in its neighborhood.
